# Development of Novel Radiopharmaceuticals for SPECT and PET

**DOI:** 10.3390/ph19060841

**Published:** 2026-05-28

**Authors:** Bianca Gutfilen

**Affiliations:** Department of Radiology, School of Medicine, Universidade Federal do Rio de Janeiro, Rio de Janeiro 21941-971, Brazil; bianca.gutfilen@gmail.com

Nuclear Medicine has experienced remarkable advances over the past few decades, driven primarily by the development of new radiopharmaceuticals for both conventional applications and positron emission tomography (PET). In this context, the concept of theranostics emerges as a cornerstone of a new era in the field, integrating diagnosis and therapy in an increasingly personalized manner.

Building on these challenges, the development of next-generation radiopharmaceuticals is not only expanding the therapeutic landscape in oncology but also opening new avenues in other clinical settings, including inflammation, infection, and regenerative medicine. Advances in radiolabeling techniques and the emergence of novel targets have enabled the exploration of increasingly specific and versatile agents, including those based on emerging radionuclides. These innovations pave the way for more precise disease characterization and therapy monitoring, as well as the integration of molecular imaging into therapeutic decision-making. Ultimately, such progress reinforces the central role of Nuclear Medicine in the era of precision medicine and highlights the potential of radiopharmaceuticals to bridge diagnosis, therapy, and biological understanding [1].

This Special Issue on the “Development of Novel Radiopharmaceuticals for SPECT and PET Imaging” aimed to explore the latest advancements in the development of novel radiopharmaceuticals for SPECT and PET imaging. This issue comprises contributions that highlight innovative synthesis techniques, novel radiotracers, improved imaging agents, and their clinical applications. Contributions address challenges and future directions, offering a comprehensive resource for enhancing SPECT and PET technologies.

In the oncologic field, recent work has highlighted the potential of radiotracers targeting B7-H3: an immune checkpoint molecule overexpressed in multiple malignancies and frequently associated with poor prognosis. To enable a noninvasive assessment of its expression for patient selection and stratification, Funeh et al. (Contribution 1) successfully labeled B7-H3-specific single-domain antibody fragments (sdAbs) with technetium-99m and evaluated their performance in xenograft tumor models. The resulting radiotracer demonstrated high tumor uptake, favorable tumor-to-background ratios, and specific binding to B7-H3-expressing tissues, supporting its utility as a sensitive imaging tool. Beyond its immediate application, this approach illustrates the broader contribution of molecular imaging in advancing targeted and immune-based therapies.

The expansion of theranostic strategies is also evident in highly challenging diseases such as glioblastoma. Kyrkou et al. (Contribution 2) developed the [^99m^Tc]Tc-AGT-7 agent, an innovative platform combining diagnostic imaging with potential therapeutic functionality. Preclinical data indicate effective tumor targeting, supporting its potential role in both visualization and treatment. This study reflects the growing interest in adaptable, target-specific theranostic agents designed to address unmet clinical needs.

Alongside tracer development, the translation of radiopharmaceuticals into clinical practice remains a critical aspect of progress in Nuclear Medicine. The technology transfer of O-(2-[^18^F]fluoroethyl)-L-tyrosine (IASOglio^®^) exemplifies the practical and regulatory efforts required to broaden access to advanced PET imaging. Notaro et al. (Contribution 3) demonstrated the feasibility of local [^18^F]FET production through a validated and automated process meeting quality and stability standards, ultimately achieving regulatory approval for clinical use in Italy. This accomplishment not only improves tracer availability but also highlights the importance of infrastructure and technology transfer in enabling timely diagnosis and management of gliomas.

Further expanding the scope of molecular imaging, novel PET probes targeting intracellular signaling pathways are being developed. Futatsugi et al. (Contribution 4) introduced [^18^F]R1487, a radiolabeled p38α inhibitor designed for noninvasive assessment of kinase activity in vivo. Preclinical evaluation revealed rapid and high uptake in brown adipose tissue, along with favorable target-to-background ratios and clear PET/CT visualization, supporting its specificity for metabolically active tissues. By enabling the imaging of p38α activation, this strategy opens new perspectives for investigating the molecular basis of inflammation, cancer, and metabolic disorders.

Targeting tumor angiogenesis through integrin αVβ3 remains a well-established strategy in molecular imaging, yet optimizing tracer pharmacokinetics continues to be a critical challenge. The development of iodine-125-labeled bicyclic RGD probes provides important insights into how radionuclide-based design can influence in vivo performance (Contribution 5). While the incorporation of albumin-binding moieties prolonged systemic circulation and increased overall tumor uptake, it did not result in a favorable tumor-to-background contrast due to sustained blood activity. In contrast, the dimeric iodine-125-labeled RGD probe achieved higher tumor accumulation along with markedly improved tumor-to-blood and tumor-to-muscle ratios, indicating the more efficient and selective targeting of αVβ3-expressing tumors. These findings emphasize the relevance of combining appropriate radionuclides with optimized molecular design and support multivalency as a particularly effective strategy for enhancing the performance of integrin-targeted radiopharmaceuticals.

In lung cancer, tumor hypoxia is a major driver of progression and treatment resistance, making its noninvasive assessment highly relevant. Alenezi et al. (Contribution 6) provide an overview of PET radiopharmaceuticals for hypoxia imaging, highlighting established tracers such as [^18^F]-FMISO alongside newer agents, including [^18^F]-FAZA, [^18^F]-HX4, and [^64^Cu]Cu-ATSM, which offer improved pharmacokinetic profiles and imaging contrast. These tracers, particularly when combined with hybrid imaging modalities, show significant potential for treatment planning and response assessment. Despite promising results, challenges related to specificity, quantification, and standardization remain, underscoring the need for continued refinement before routine clinical implementation.

In parallel, dual-modality imaging strategies are gaining increasing attention for their potential to integrate preoperative diagnostics with intraoperative guidance. Gariglio et al. (Contribution 7) developed two [^68^Ga]-labeled PET/fluorescence probes targeting the cholecystokinin-2 receptor (CCK2R), demonstrating high receptor affinity, efficient internalization, and specific tumor uptake in preclinical models. Although both probes enabled clear tumor visualization with reduced off-target accumulation, differences in renal retention highlight the impact of chelator selection on in vivo performance. These findings further support the clinical potential of dual-modality radiopharmaceuticals in improving tumor detection and surgical guidance.

Extending beyond oncology, Cunha et al. (Contribution 8) provided preliminary evidence supporting the use of [^99m^Tc]Tc-anti-TNF-α scintigraphy for assessing inflammatory activity in hidradenitis suppurativa. In addition to confirming uptake in clinically evident lesions, this study suggests the detection of previously unrecognized inflammatory sites, indicating a potential advantage over conventional clinical evaluation. Although limited by its pilot design, this study points to the value of targeted molecular imaging in refining disease assessment in inflammatory conditions.

Taken together, these advances illustrate a rapidly evolving landscape in Nuclear Medicine, driven not only by the continuous development of novel radiopharmaceuticals but also by improvements in molecular design, multimodal imaging, and translational implementation. From target-specific tracers and intracellular pathway imaging to dual-modality probes and strategies to enhance clinical accessibility, the field is moving toward increasingly precise, personalized, and clinically impactful applications. At the same time, persistent challenges, including optimizing pharmacokinetics, standardizing imaging protocols, and ensuring broader availability of advanced tracers, must be addressed to allow the full translation of innovations into routine practice. Ultimately, the integration of these approaches reinforces the central role of Nuclear Medicine in precision medicine, positioning radiopharmaceuticals as important tools not only for diagnosis and therapy but also for gaining a deeper understanding of complex biological processes across a wide spectrum of diseases.
